# Freehand Stereotactic Image-Guidance Tailored to Neurotologic Surgery

**DOI:** 10.3389/fsurg.2021.742112

**Published:** 2021-10-07

**Authors:** Daniel Schneider, Lukas Anschuetz, Fabian Mueller, Jan Hermann, Gabriela O'Toole Bom Braga, Franca Wagner, Stefan Weder, Georgios Mantokoudis, Stefan Weber, Marco Caversaccio

**Affiliations:** ^1^ARTORG Center for Biomedical Engineering, University of Bern, Bern, Switzerland; ^2^Department of Otorhinolaryngology, Head & Neck Surgery, Inselspital, University Hospital Bern, Bern, Switzerland; ^3^Department of Diagnostic and Interventional Neuroradiology, Inselspital, University Hospital Bern, Bern, Switzerland

**Keywords:** clinical trial, neurotology, lateral skull, lateral skull base, temporal bone, image-guidance, surgical navigation, accurate navigation in neurotology

## Abstract

**Hypothesis:** The use of freehand stereotactic image-guidance with a target registration error (TRE) of μ_TRE_ + 3σ_TRE_ < 0.5 mm for navigating surgical instruments during neurotologic surgery is safe and useful.

**Background:** Neurotologic microsurgery requires work at the limits of human visual and tactile capabilities. Anatomy localization comes at the expense of invasiveness caused by exposing structures and using them as orientation landmarks. In the absence of more-precise and less-invasive anatomy localization alternatives, surgery poses considerable risks of iatrogenic injury and sub-optimal treatment. There exists an unmet clinical need for an accurate, precise, and minimally-invasive means for anatomy localization and instrument navigation during neurotologic surgery. Freehand stereotactic image-guidance constitutes a solution to this. While the technology is routinely used in medical fields such as neurosurgery and rhinology, to date, it is not used for neurotologic surgery due to insufficient accuracy of clinically available systems.

**Materials and Methods:** A freehand stereotactic image-guidance system tailored to the needs of neurotologic surgery–most importantly sub-half-millimeter accuracy–was developed. Its TRE was assessed preclinically using a task-specific phantom. A pilot clinical trial targeting N = 20 study participants was conducted (ClinicalTrials.gov ID: NCT03852329) to validate the accuracy and usefulness of the developed system. Clinically, objective assessment of the TRE is impossible because establishing a sufficiently accurate ground-truth is impossible. A method was used to validate accuracy and usefulness based on intersubjectivity assessment of surgeon ratings of corresponding image-pairs from the microscope/endoscope and the image-guidance system.

**Results:** During the preclinical accuracy assessment the TRE was measured as 0.120 ± 0.05 mm (max: 0.27 mm, μ_TRE_ + 3σ_TRE_ = 0.27 mm, *N* = 310). Due to the COVID-19 pandemic, the study was terminated early after *N* = 3 participants. During an endoscopic cholesteatoma removal, a microscopic facial nerve schwannoma removal, and a microscopic revision cochlear implantation, *N* = 75 accuracy and usefulness ratings were collected from five surgeons each grading 15 image-pairs. On a scale from 1 (worst rating) to 5 (best rating), the median (interquartile range) accuracy and usefulness ratings were assessed as 5 (4–5) and 4 (4–5) respectively.

**Conclusion:** Navigating surgery in the tympanomastoid compartment and potentially in the lateral skull base with sufficiently accurate freehand stereotactic image-guidance (μ_TRE_ + 3σ_TRE_ < 0.5 mm) is feasible, safe, and useful.

**Clinical Trial Registration:** www.ClinicalTrials.gov, identifier: NCT03852329.

## Introduction

The temporal bone anatomy is unique in the human body with a geometric size in the submillimeter range (1). In addition, the homogenously colored and structured bone obscures anatomical structures to the human eyes and tactile sensations. Hence, neurotologic microsurgery requires surgeons to operate at the limits of their visual and tactile capabilities, safe instrument guidance is challenging, and experience is paramount to a safe procedure. To maximize surgical safety during neurotologic surgery, structures that would ideally be preserved are exposed sequentially based on their expected spatial locations and arrangement. The exposed structures are then used as landmarks to infer the instruments' location in the context of the anatomy and used to guide the surgical procedure. While this manual visuospatial localization method is standard in neurotologic surgery, (1) it is inherently invasive as instrument localization demands overexposure of the surgical *situs* to be safe (i.e., invasiveness is the burden accepted for the localization of anatomy and instruments), and (2) its localization accuracy is dependent on experience and anatomy, i.e., the more abnormal the anatomy, the lower the localization accuracy and precision.

There exists an unmet clinical need for sub-half-millimeter accurate and precise anatomy localization and instrument navigation during neurotologic surgery to reduce invasiveness and potentially avoid harm to critical anatomical structures.

During the 1980s, electromyography monitoring of the facial nerve and stereotactic image-guidance, were explored as potential solutions to this longstanding unmet need. In the last 30 years, facial nerve monitoring has proven valuable and is routinely used to map the facial nerve (2). However, after 30 years of intensive preclinical and clinical research on freehand stereotactic image-guidance, other surgical domains such as sinus surgery (3) and neurosurgery (4) benefit from the standard application of the technology. Despite the clinical availability of systems with negligible system-associated costs, freehand stereotactic image-guidance is not routinely used in neurotologic surgery (5). Custom (6) and commercially (7–13) available freehand stereotactic image-guidance systems were used in numerous clinical research applications. The technology was reported as useful for identifying anatomy and pathology in (pseudo)neoplasm resection (7, 8, 13), cochlear implantation (9, 10), congenital aural atresia (12), and auricular implant (14) surgery but no significant effects on safety or efficacy were demonstrated. Other attempts have suggested potential usefulness in otologic, petrous apex, and internal auditory canal surgery (6, 11, 15, 16). The persistent lack of proven effects and absence in routine use, however, indicate that the clinically available freehand image-guidance systems still do not meet the needs of neurotologic surgery, most importantly the need for sub-half-millimeter localization accuracy and precision.

The technical challenge of providing image-guidance with an accuracy <0.5 mm (μ_TRE_ + 3σ_TRE_) has been solved to enable minimally-invasive cochlear implantation using a keyhole approach (17, 18). Safe image-guided creation of a keyhole middle and inner ear access effective for cochlear implantation requires an image guidance error <0.5 mm (μ_TRE_ + 3σ_TRE_) (19). Clinical studies using microstereotactic frames (20) and an image-guided robot (21, 22) demonstrated that an accuracy <0.5 mm (μ_TRE_ + 3σ_TRE_), required for safe cochlear access (19), can be provided in a clinical setting. By now, a task-autonomous image-guided robot indicated for use in cochlear implantation (HEARO®, CASCINATION AG, Switzerland) is available on the market. It delivers the required performance characteristics, enabling safe creation of a middle and inner ear access effective for cochlear implantation.

Analogously, for the safe use of freehand stereotactic image-guidance in the sub-half-millimeter sized temporal bone anatomy, the localization accuracy and precision must be proportionate, i.e., the target registration error (TRE) (23) must be such that μ_TRE_ + 3σ_TRE_ <0.5 mm.

Today, no clinically available freehand stereotactic image-guidance system provides a localization accuracy and precision within this range sufficient for neurotologic surgery.

Here we present a clinical study with a freehand stereotactic image-guidance system tailored to neurotologic surgery for which the accuracy was preclinically verified to be <0.5 mm (μ_TRE_ + 3σ_TRE_). The objective assessment of quantitative instrument localization errors <0.5 mm (μ_TRE_ + 3σ_TRE_) is impossible in a clinical setting because establishing a sufficiently accurate ground-truth is impossible. Therefore we herein propose and applied a method to clinically validate the accuracy of freehand stereotactic image-guidance based on surgeons' ratings of corresponding image pairs from the microscope and the image-guidance system.

## Methods

### A System Tailored to Neurotologic Surgery

**Figure 1:**
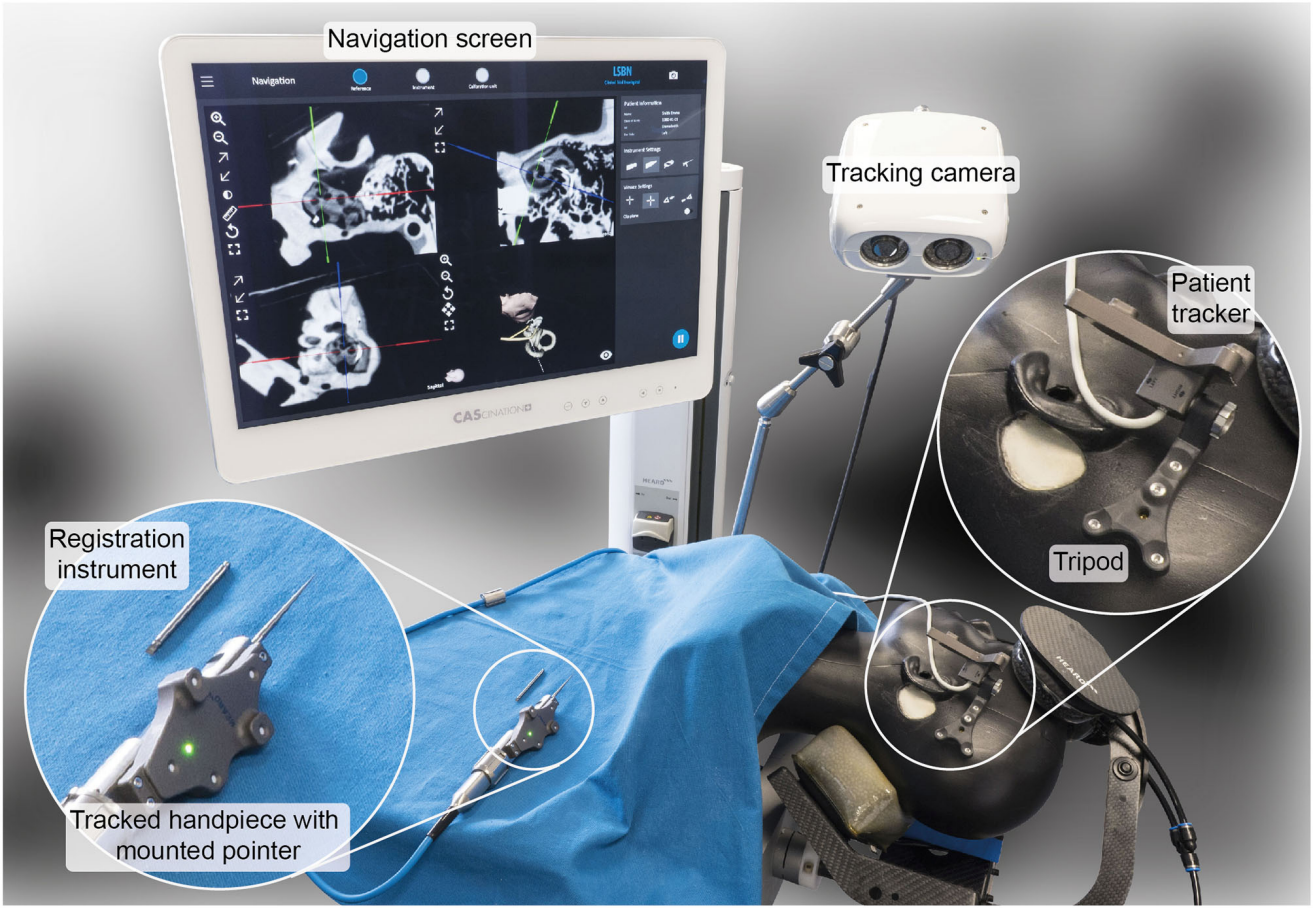
Freehand stereotactic image-guidance system tailored to neurotologic surgery. It comprises a spatial tracking camera, a tracked handpiece providing the possibility to track a registration and pointer instrument, a bone-anchored tripod embedding four titanium registration fiducials and providing an interface to attach the patient tracker, and a navigation user interface.

Based on image-guidance technology developed for robotic cochlear implantation with proven accuracy μ_TRE_ + 3σ_TRE_ = 0.39 mm (21), a freehand stereotactic image-guidance system tailored to neurotologic surgery, i.e., sufficient accuracy and precision (μ_TRE_ + 3σ_TRE_ <0.5 mm), and compatibility in transmastoidal, transfacial recess, transmeatal, transtympanic, and transpetrosal approaches to the internal auditory canal, petrous apex, middle and inner ear - was implemented (Figure 1).

A spatial tracking system with a maximum measurement error ≤ 0.05 mm was used (CamBar B1, Axios 3D Services, Germany). A tracked handpiece embedding an instrument mount enabled the tracking of a registration (length: 30 mm, tip diameter: 2.8 mm) and a pointer (length: 60 mm, tip diameter: of 0.4 mm) instrument. The instruments' geometries were preoperatively calibrated.

A tripod was developed that combines bone-anchored registration fiducials and rigid attachment of the patient tracker to the patient in a single structure. The tripod body was made of radio-translucent carbon-reinforced polyether ether ketone (PEEK). It embeds four titanium registration fiducials, three of which contact the skull. Additionally, the template provided an interface to attach the patient tracker. The tripod structure was rigidly fixed to the skull with a bone screw.

A portable CBCT scanner designed for intraoperative use was used for imaging (resolution: 0.2 mm^3^; XCAT, Xoran Technologies, USA) in combination with a metal-free horse-shoe headrest (Maquet, Germany). To annotate the temporal bone, facial nerve, chorda tympani, ossicles, cochlea, and the ear canal in the image data and compute 3D reconstructions thereof, OTOPLAN® (CASCINATION AG, Switzerland) was used. The positions of the titanium registration fiducials are automatically detected in OTOPLAN®.

The processed image data was fed into a custom navigation software that superimposes the location of the tracked instruments on top.

### Preclinical TRE Assessment

**Figure 2:**
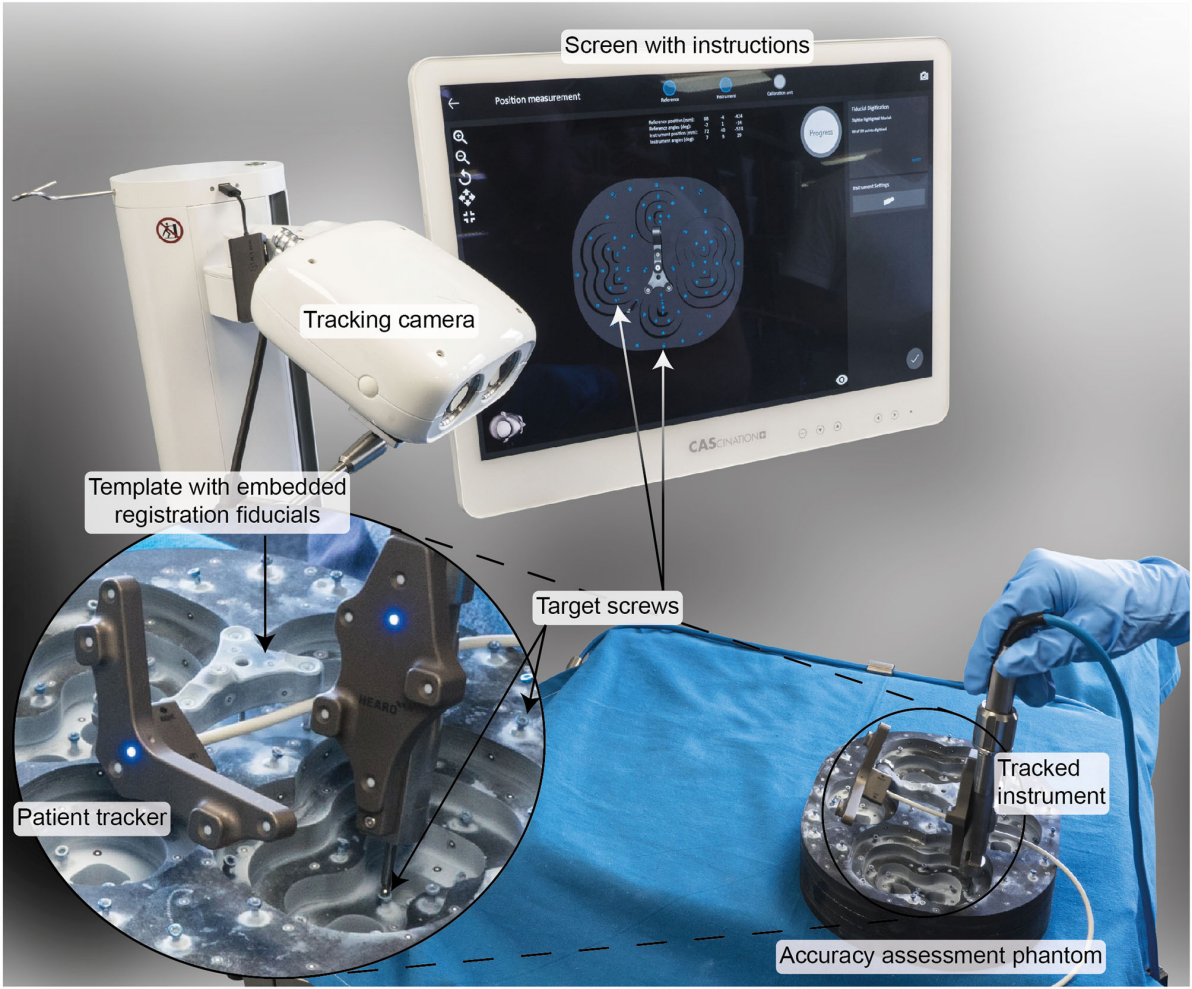
Experimental setup to preclinically verify a TRE < 0.5 mm (μ_TRE_+3σ_TRE_). A task-specific phantom made of carbon fiber and measured using a coordinate measurement machine with a measurement error <0.015 mm was used. The user was guided by the software through the registration and measurement process.

In a technical preclinical setting, the TRE of the developed system was assessed using a task-specific phantom complying with the requirements from the standard for measurement of positional accuracy of computer assisted surgical systems [ASTM F2554 (24), Figure 2]. The phantom consists of a carbon-fiber structure mimicking the left and right temporal bone anatomy. The carbon material assures negligible deformation due to acting forces and temperature changes. The registration tripod is fixed on the structure using a bone screw. Sixty-two titanium screws were fixed on the carbon structure and serve as target structures. The geometry of the phantom was measured with a coordinate measurement machine (CMM, measurement error ≤ 0.015 mm; dim Dienste Industrielle Messtechnik GmbH, Germany). The phantom thereby allows measurement of the TRE of a freehand stereotactic image-guidance system with a measurement accuracy ≤ 0.015 mm and is thus suitable for assessing the errors of freehand stereotactic image-guidance systems down to ~0.1 mm [ASTM F2554 (24)].

During the experiments, the patient tracker was attached to the tripod structure. The positions of the registration fiducials embedded in the tripod template and all 62 target screws were measured five times by three study participants. The tracking measurements were registered to the CMM measurements using the registration fiducials. The TRE was calculated for each target screw.

To include error contributions from imaging in the TRE measurements, a CBCT image (resolution: 0.2 mm^3^; XCAT, Xoran Technologies, USA) was acquired. In the image data, the registration fiducials and target screws were automatically detected using OTOPLAN^®^ (CASCINATION AG, Switzerland). The tracking measurements were registered to the image data using the registration fiducials. The end-to-end system TRE (inclusive error contributions from imaging) was calculated at each target screw. To this end, the CMM measurements were registered to the detected target screw positions and used as ground truth.

Mean, standard deviation, and maximum TRE values were calculated.

### Clinical Accuracy and Usefulness Validation

#### Study Design

A prospective, single-arm, noncontrolled pilot clinical trial targeting *N* = 20 study participants between January and July 2020 was planned (KEK 2019-00128, Swissmedic 10000563, ClinicalTrials.gov ID: NCT03852329) to validate the accuracy and usefulness of the developed system during neurotologic surgery.

The primary objective of the clinical trial was to validate the image-guidance accuracy, knowing that the establishment of a sufficiently accurate ground truth and thus the objective measurement of the quantitative error is infeasible in a clinical setting. A method based on accuracy and usefulness ratings of corresponding microscope and image-guidance views of multiple surgeons was proposed and used for this study. The secondary objective was to evaluate the usefulness of the information obtained from the freehand stereotactic image-guidance system.

Participant recruitment started 01.01.2020 and included full-aged, non-pregnant patients, which were regularly scheduled for an otologic or neurotologic surgical procedure. Informed consent was obtained at the latest during hospital admission the day before the surgery.

#### Intraoperative Workflow

The complete intervention was performed under general anesthesia.

##### Patient Preparation and System Setup

The participant was prepared for the surgery according to clinical standards. The navigation system was set up concurrently. The navigation platform was positioned next to the operating table opposite to the surgeon. The spatial tracking camera was fixed on the operating table and connected to the platform. The microscope or endoscope video stream was rerouted to the navigation platform.

##### Tripod Fixation

The tripod with embedded registration fiducials was fixed to the skull supero-posterior to the ear canal using one bone screw. To this end, the site was prepared with local anesthetics and four stab incisions, one for the bone screw, and three for the tripod legs contacting the bone. A pilot hole was drilled using a drill (OsteoDriver 2, OsteoMed, USA) with a mechanical stop at 4 mm.

##### CBCT Imaging

Subsequently, a CBCT image was acquired in the operating room (resolution: 0.2 mm^3^; XCat, XORAN Technologies, USA).

##### Conventional Surgical Treatment

Thereafter, the surgical procedure proceeded according to clinical standards.

##### Image Annotation

Simultaneously, the image data were preprocessed using OTOPLAN^®^ (CASCINATION AG, Switzerland). The registration fiducials were automatically detected and the temporal bone, facial nerve, chorda tympani, cochlea, malleus, incus, stapes, and ear canal were semi-automatically segmented. The semi-circular canals were manually segmented using Amira (Thermo Fischer Scientific, USA). The image data and extracted information were transferred to the navigation platform.

##### Start Navigation

The surgical procedure was interrupted for a few minutes. The navigation software was started. The patient tracker was attached to the tripod and the tracking camera's orientation was adjusted toward the patient. The registration instrument was mounted in the handpiece and the surgeon was guided through the registration procedure by the navigation software. Thereafter, the navigation software superimposed the tracked instrument position on the preprocessed image data.

##### Data Collection

**Figure 3:**
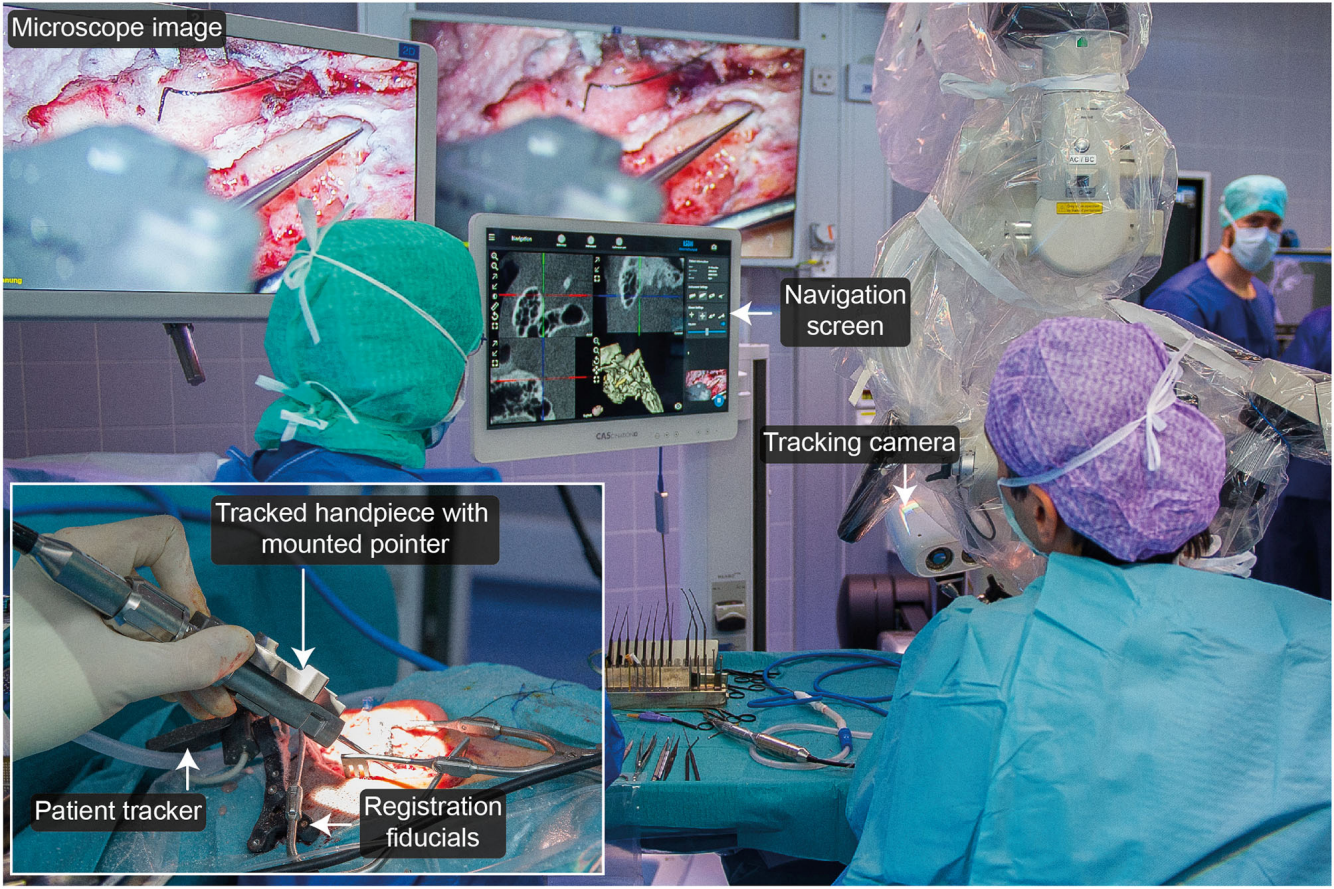
Experimental setup during microscopic removal of a facial nerve schwannoma.

After registration, the surgical procedure was continued according to clinical standards. Whenever a suitable landmark was exposed, the pointer was positioned on the landmark (Figure 3) and, at the press of a button, the navigation software saved a screenshot of the microscope/endoscope stream and the pointer's pose information. To comply with the standard surgical treatment, the anatomical target landmarks to be used were defined for each procedure separately and included the spine of Henle, incus (short process), facial nerve, dura, sigmoid sinus, lateral semi-circular canal, round window, oval window, stapes (anterior and posterior arches), eustachian tube (entrance), and Notch of Rivinus.

During the procedure, the timing of the individual workflow steps was measured.

#### Postoperative Workflow

Postoperatively, the virtual navigation scene was reconstructed for each anatomical landmark and the view direction in the 3D viewer was manually aligned with the microscope image. Screenshots of the MPR viewer and the 3D view were stored in a locally hosted database together with the corresponding microscope or endoscope image for each landmark and participant. The data was made accessible via a password-protected website showing the microscope/endoscope images and corresponding 3D views and MPR slices from the navigation system for each participant and landmark. The website provided functionality to give an accuracy and usefulness rating per participant and landmark. Five surgeons received individual login data and were asked to grade the image-guidance accuracy using the following 5-level (Strongly agree, Agree, Undecided, Disagree, Strongly disagree) Likert item:

The position indicated by the navigation view is correct on a clinically relevant geometric scale.

Furthermore, the five surgeons were asked to rate the usefulness of the displayed information with the following 5-level (Strongly agree, Agree, Undecided, Disagree, Strongly disagree) Likert item:

The information provided by the navigation view is useful to identify the anatomical structure.

#### Endpoints

The primary and secondary endpoints were the median (and interquartile range) accuracy and usefulness rating, respectively. An additional secondary endpoint was the timing of the individual surgical and system-associated steps.

## Result

### Preclinical TRE Assessment

The TRE of the stereotactic system without contributions from imaging was 0.120 ± 0.05 mm (max: 0.27 mm, μ_TRE_ + 3σ_TRE_= 0.27 mm, *N* = 310). The end-to-end TRE was 0.150 ± 0.06 mm (max: 0.31 mm, μ_TRE_ + 3σ_TRE_ = 0.34 mm, *N*= 310).

### Clinical Validation

Twelve study participants were screened, three were enrolled in the study, one declined to take part, and 8 eight tentative candidates had to be canceled due to the COVID-19 pandemic.

**Figure 4:**
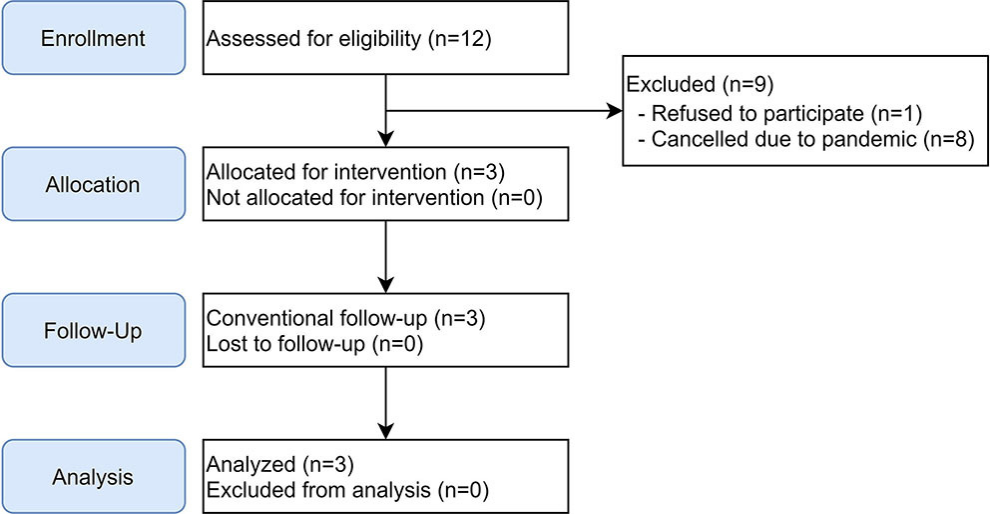
Flowchart for non-randomized clinical trial.

Participant recruitment was suspended on the 16.03.2020 due to the COVID-19 pandemic. The study was terminated early in June 2020 after three enrolled and completed subjects. Available results are presented and discussed in the following paragraphs (Figure 4).

The study included an endoscopic cholesteatoma removal, a microscopic removal of a facial nerve schwannoma in the mastoid segment, and a microscopic revision cochlear implantation (Table 1). The study-specific procedure was successfully conducted in two of the three participants. The subject scheduled for a revision cochlear implantation showed scarring and adhesion of subcutaneous tissue. During manipulation of the tissue, the tripod structure with the embedded registration fiducials became loose and eventually fell off. The operation was successfully completed without primary endpoint data collection.

**Table 1:** Study participants and their respective indications for surgery, access routes, visualization techniques, and whether study-related data acquisition was completed.

**Participant**	**Pathology**	**Surgical access**	**Visualization technique**	**Data acquisition complete**
1	Cholesteatoma	Transmeatal	Endoscopic	✓
2	Facial nerve schwannoma	Transmastoidal	Microscopic	✓
3	Revision cochlear implant	Transmastoidal and trans-facial recess	Microscopic	Partially

**Figure 5:**
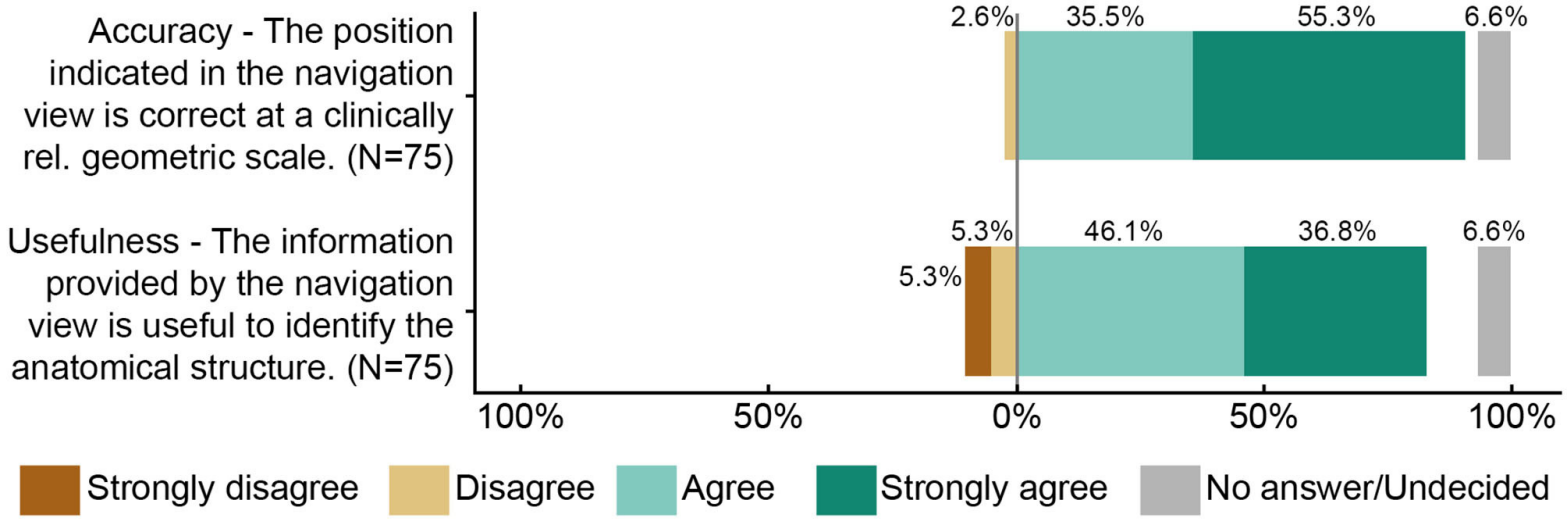
Summary of the accuracy and usefulness ratings (*N* = 75). Five surgeons each rated 15 anatomical landmarks from two study participants.

**Figure 6:**
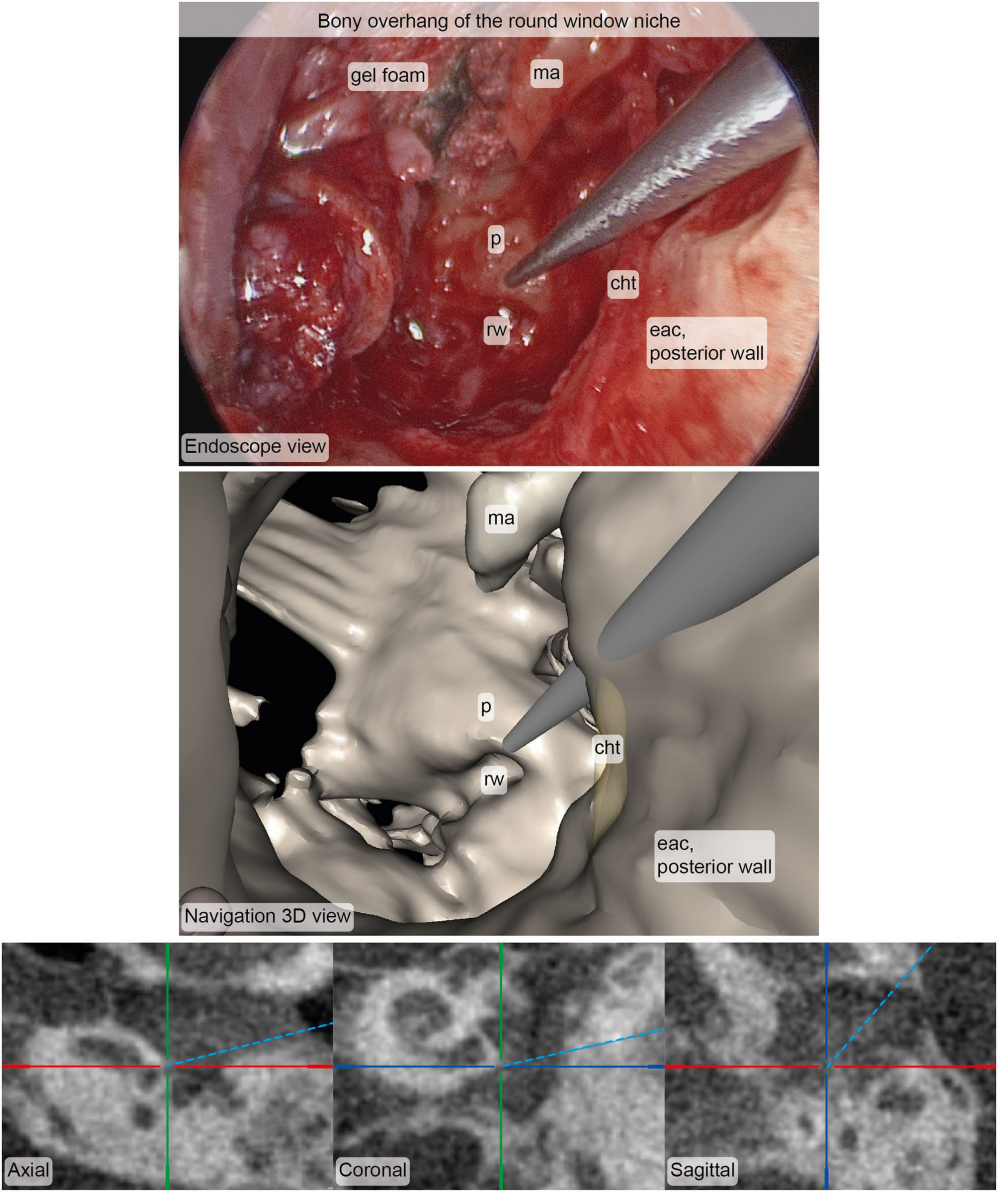
Example of corresponding images from the endoscope (top) and the stereotactic image-guidance system (bottom) depicting the pointer pose at the bony overhang of the round window niche. The image pair was rated in terms of accuracy (median: 4, interquartile range: 4–5, *N* = 5) and usefulness (median: 4, interquartile range: 4–5, *N* = 5). Inspection of and zooming into the individual MPR and 3D viewer panels were possible during rating. cht, chorda tympani; eac, external auditory canal; ma, malleus; p, promontory; rw, round window.

**Figure 7:**
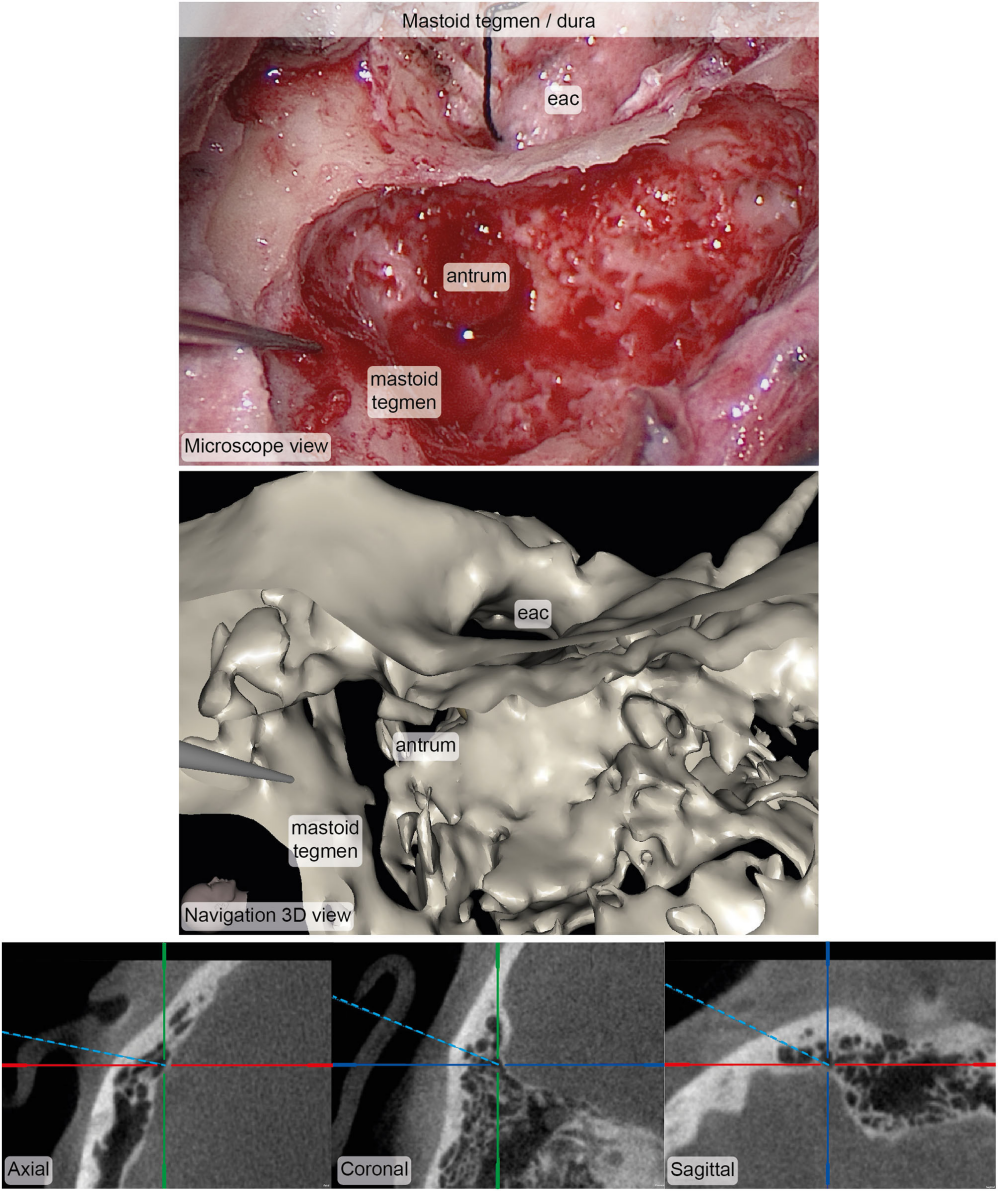
Example of corresponding images from the microscope (top) and the stereotactic image-guidance system (bottom) depicting the pointer pose on the bone of the mastoid tegmen covering the dura. The image pair was rated in terms of accuracy (median: 4, interquartile range: 3.5–4.5, *N* = 5) and usefulness (median: 4, interquartile range: 4–4.5, *N* = 5). Inspection of and zooming into the individual MPR and 3D viewer panels were possible during rating. eac, external auditory canal.

Primary endpoint measurements at a total of 15 anatomical landmarks (ten and six for the first and second participants, respectively) were acquired and postprocessed for the survey. Five surgeons filled out the survey, resulting in N = 75 ratings. On a scale from 1 (worst rating) to 5 (best rating), the median (interquartile range) accuracy and usefulness ratings were assessed as 5 (4–5) and 4 (4–5) respectively (Figure 5). In more than 90% of the ratings, the surgeon agreed or strongly agreed that the position estimate of the stereotactic image-guidance accuracy was correct on a clinically relevant scale. In more than 80% of the ratings, the surgeon agreed or strongly agreed that the information provided by the navigation view is useful for the surgical task at hand. Two example pairs of corresponding images from the microscope/endoscope and the stereotactic image-guidance system are depicted in Figures 6, 7. All pairs of images and associated ratings can be inspected in the attachment.

**Figure 8:**
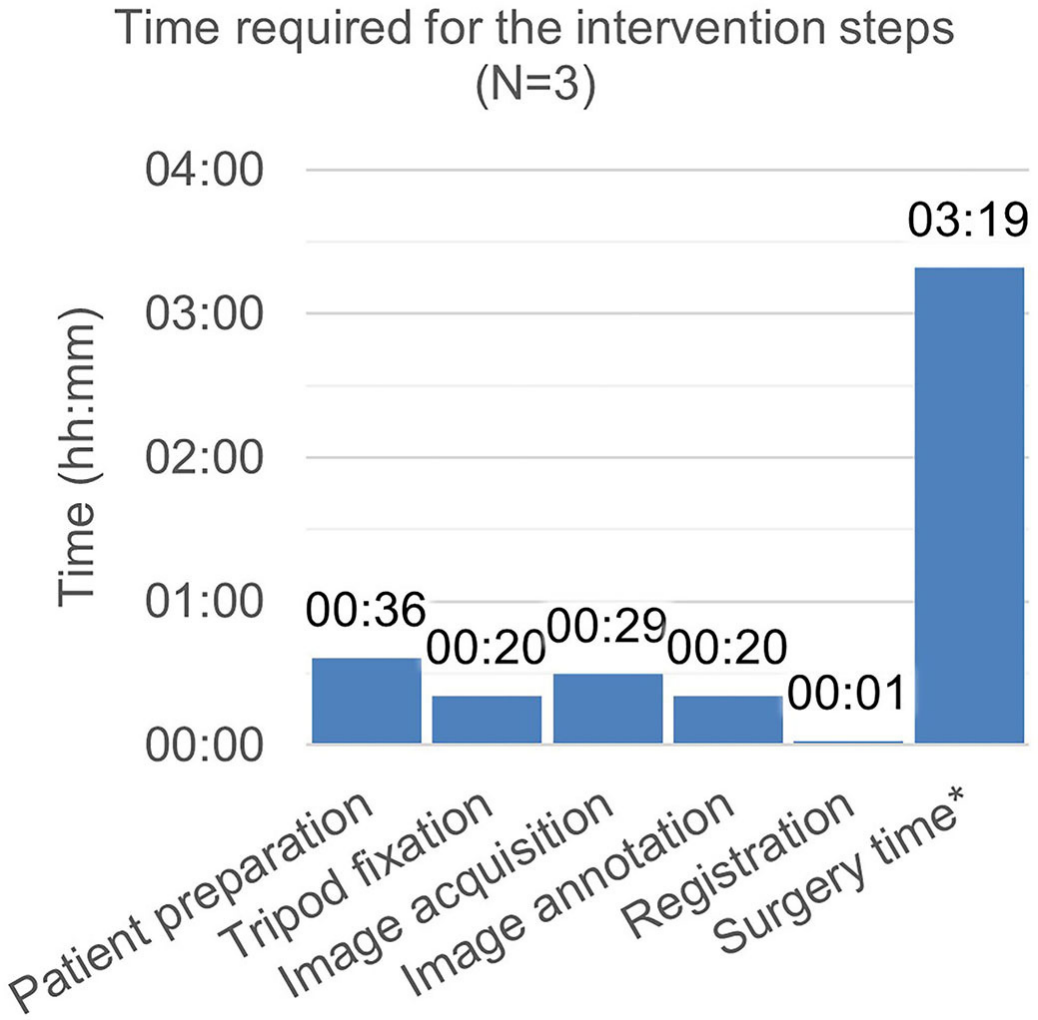
Time required for the intervention steps. *Surgery time includes the non–study-related time during which period, the surgeon manipulates the patient. Time measurement was started with the incision and ended with the finished suture. It includes image annotation time (as the surgery was simultaneously continued). It excludes time required for image acquisition and export, as, during this period, the surgery was interrupted.

The time expenditure for the individual workflow steps during the intervention is depicted in Figure 8. The surgery time prolongation caused by study-specific interventions was 52 min and the total time of study-specific interventions amounted to 78 min (image annotation was conducted in parallel with the surgical treatment).

## Discussion

This work presents a freehand stereotactic image-guidance system tailored to neurotologic surgery. The system was designed to enable an instrument localization error <0.5 mm (μ_TRE_ + 3σ_TRE_). Its accuracy was successfully verified using a task-specific preclinical technical accuracy phantom complying with the requirements from the standard for measurement of positional accuracy of computer assisted surgical systems [ASTM F2554 (24)]. Finally, a method based on subjective surgeon ratings of corresponding microscope/endoscope and navigation image pairs was proposed and used to validate clinically, the accuracy and usefulness of the device.

### Accuracy Validation

There have been various preceding clinical studies using freehand stereotactic image-guidance during neurotologic surgery (5). A common study endpoint is the TRE. The effective clinical TRE is highly relevant because it describes the reliability of the information presented by the system and thus dictates whether the utilization of a system is safe and potentially effective. The standard method for assessing the TRE in a clinical setting is to measure the distance between the instruments' position as indicated by the image-guidance system and the actual instrument position as identified by visual inspection with the naked eye (7, 12, 13, 25–32). However, it is impossible to objectively identify the true instrument position in image data by visual inspection with the naked eye with the precision required to measure sub-half-millimeter image-guidance errors (required measurement error ≲0.05 mm). The only effort to devise an alternative method for clinical accuracy validation of freehand stereotactic image-guidance was made by Balachandran et al. They proposed to measure the TRE during BAHA® surgery using the BAHA® bone screw as target fiducial (33). While this method allows assessment of the quantitative error, the authors themselves state that it is limited to assessment on the surface of the temporal bone and therefore of limited relevance to neurotology. Other preclinically used measurement methods, such as the measurement of implanted target screws (34–37) or the geometric comparison of a planned surgical approach vs. its image-guided execution (17, 18, 38), are not ethically justifiable for use *in vivo* or require an enormous effort rendering the achievement of statistical significance infeasible.

Thus, in a neurotological clinical setting, objective determination of the quantitative image-guidance error of freehand stereotactic image-guidance is impossible; especially by visual inspection with the naked eye.

The TRE of a freehand stereotactic image-guidance system dedicated to neurotologic surgery must be determined preclinically on a technical task-specific phantom. Following this, the safety and usefulness of the device must be clinically investigated whereby the image-guidance error can only be subjectively validated. Herein, a method to validate clinically the image-guidance error of freehand stereotactic image-guidance based on subjective surgeon ratings is proposed. By acquiring image pairs from corresponding surgical (microscope/endoscope) and navigation views and collecting associated accuracy ratings from multiple surgeons, accuracy validation is achieved through intersubjectivity assessment. A quantitative ground truth is not required. Furthermore, the method allows for comprehensibility and transparency through the publication of corresponding pairs of images and ratings and is scalable in sample size as the evaluation is conducted postoperatively. To avoid confirmation bias in the evaluation, the rating must be performed by surgeons who are independent of the project that involves the clinical study.

### Accuracy Requirements

Qualitative discussions on accuracy requirements for image-guidance in neurotologic surgery reported maximum values of 0.5 mm (39) and 1 mm (9, 27, 40, 41). Quantitative accuracy requirements for safe keyhole access via a posterior tympanotomy to the middle ear were calculated. With an image-guidance error of 0.39 mm (μ_error_ + 3σ_error_) and a tool diameter of 1.8 mm, 47% of the adult population can be treated safely (19). This analysis suggests that the accuracy requirements for the safe application of image-guidance in neurotologic surgery are even stricter than μ_TRE_ + 3σ_TRE_ <0.5 mm. The accuracy requirement of 0.5 mm (μ_TRE_ + 3σ_TRE_), in our opinion, presents an upper limit above which the application of image-guidance is neither safe nor effective for use in neurotologic surgery.

Clinically assessed TRE values (μ_TRE_ + 3σ_TRE_) of freehand stereotactic image-guidance reported in the literature range from 0.92 mm (6) up to 8.3 mm (25) with many values in the 1–4 mm range (7, 8, 12, 13, 26–33). Of those who specified their measurement methodology, 12 of 13 used visual inspection to determine the TRE (6, 7, 12, 13, 25–32) [the other used BAHA^®^ posts as target in six subjects (33)]. The reported TRE values are not only above the TRE limit of 0.5 mm (μ_TRE_ + 3σ_TRE_), but also require extremely critical consideration. As previously discussed, in a clinical application it is impossible to objectively assess the quantitative TRE for freehand stereotactic image-guidance dedicated to neurotologic surgery.

Potential reasons for the lack of accuracy are inaccurate spatial tracking systems, imaging with limited resolution, and inaccurate registration methods (5). The previously mentioned studies used (1) tracking cameras with a maximum spatial tracking error of 0.2–0.5 mm (Polaris systems, NDI, Canada) (6–10, 16, 25, 27, 29–31, 33, 42–47), (2) imaging with a slice thickness of 0.3 mm (48), 0.4 mm (49, 50) or higher (6–10, 12–14, 16, 25–30, 32, 33, 44–47, 51), and (3) registration based on paired-point matching using anatomical (8, 10, 13, 27, 28, 36, 47) or skin-affixed landmarks (7, 11, 14, 27, 28, 30, 42, 43, 51), or surface matching (12, 13). Unlike in those studies, an image-guidance system using a spatial tracking system with a maximum tracking error <0.05 mm, imaging with a slice thickness of 0.2 mm, and registration based on bone-anchored titanium fiducials were used.

During the last 30 years, various studies have been conducted using systems with negligible system-associated costs, but these have not seen uptake for neurotologic procedures, because of insufficient accuracy (5). However, few clinical studies have been conducted to investigate and possibly eventually exploit the benefits of sufficiently accurate freehand stereotactic image-guidance (μ_TRE_+3σ_TRE_≲ 0.5 mm) (20–22). To date, providing stereotactic image-guidance with sub-half-millimeter accuracy was achieved only using bone-anchored fiducials for registration, intraoperative imaging with a slice thickness <0.2 mm, and spatial tracking systems with a maximum tracking error <0.05 mm (20–22). Fiducials anchored in bone require incisions and time for fixation. Intraoperative imaging prolongs anesthesia time and increases radiation exposure for the patient. The CBCT scanner used for this study added uses 0.274 mSv per image (120 kVp). Image acquisition prolonged the surgery time by approximately 20–30 min. Although the costs for such a system are higher compared with clinically available freehand stereotactic image-guidance systems, the benefit-cost ratio could improve by realizing the clinical impact of the technology on invasiveness and iatrogenic injury.

### Usefulness Assessment

Significant benefit of freehand stereotactic image-guidance for neurotologic surgery has not been demonstrated to date (5). However, various studies reported potential usefulness and thereby corroborate an unmet clinical need for accurate and precise instrument localization in neurotologic surgery (5). Based on intraoperative judgement by the operating surgeon potential usefulness during surgical access creation to the petrous apex (7, 11, 13, 27, 52) and to the internal auditory canal (16, 27, 43, 52), resection of (pseudo)neoplasms (27, 52, 53), congenital bony aural atresia surgery (12), cochlear implantation (6, 9, 10) and revision surgery (27) was reported. In the presented study, during the removal of the facial nerve schwannoma, the image-guidance system was subjectively judged useful by the operating surgeon, mostly to delineate the facial nerve and determine the instrument's proximity to the dura. However, much more importantly, the herein presented method allows to validate the usefulness of a freehand image-guidance system through intersubjectivity assessment of surgeons, resulting in a more objective usefulness statement.

### Endoscopic Neurotologic Surgery

**Figure 9:**
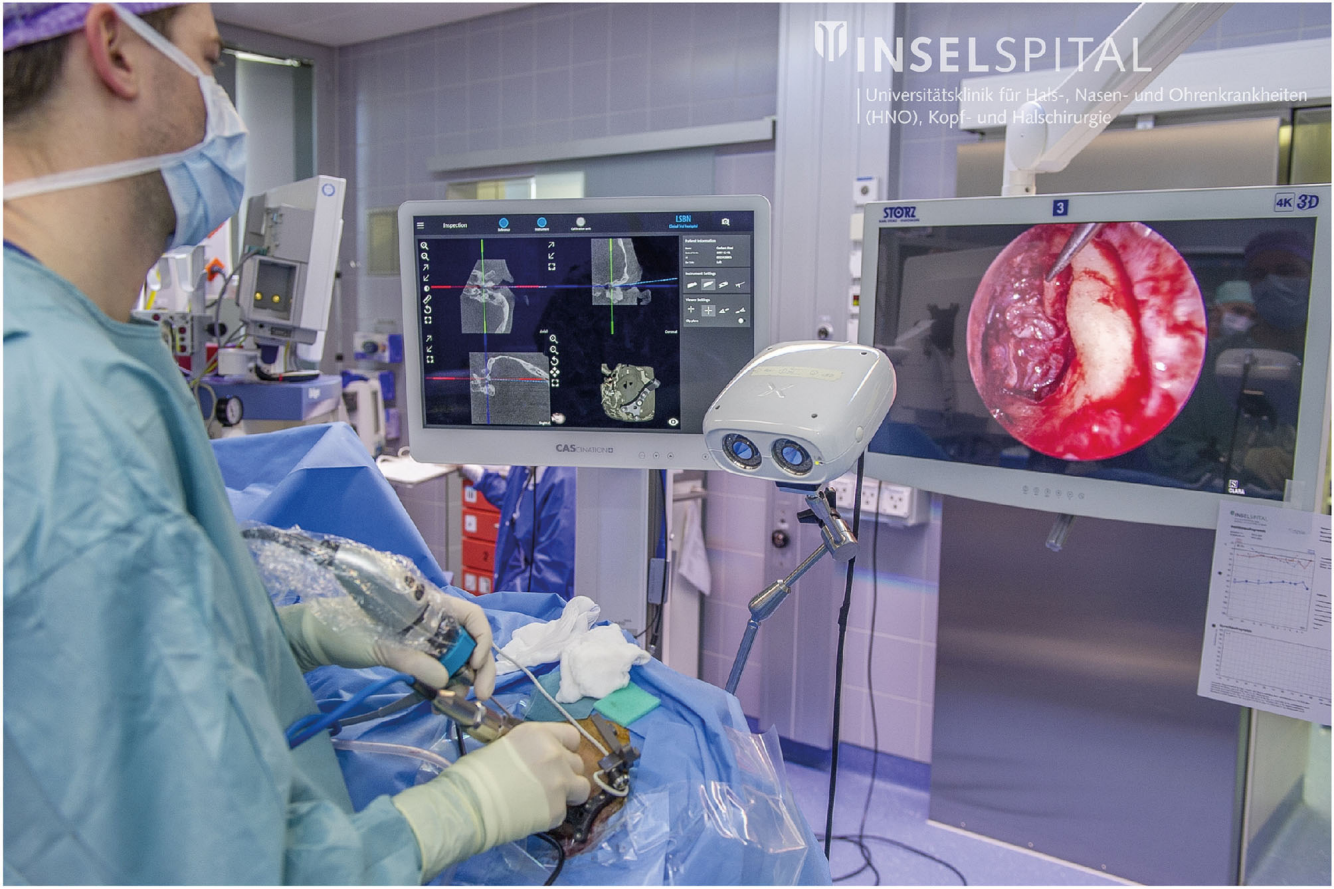
Freehand stereotactic image-guidance during an endoscopic cholesteatoma removal.

We present the first clinical application of freehand stereotactic image-guidance during endoscopic neurotologic surgery. Its use was investigated in preclinical studies by Kempfle et al. (54) and Rathgeb et al. (35). Both agreed that the possibility to see below the surface using the image-guidance system adds valuable information to the 2-dimensional perception of the anatomy from the endoscope. This additional visual feedback can be useful to intraoperatively optimize the surgical approach and exposure for effective treatment while safe instrument navigation is provided by image-guidance. The image-guidance system presented in this article seamlessly integrated during the endoscopic removal of the cholesteatoma. The developed tripod providing bone-anchored registration fiducials and rigid patient tracker attachment enables accurate and precise stereotactic image-guidance also in minimally invasive endoscopic neurotologic surgery at the cost of four stab incisions. The navigation screen was installed next to the screen showing the endoscope video. This enabled the surgeon to obtain information from the navigation system without losing sight of the instruments in the endoscopy video (Figure 9).

### Template-Embedded Registration Fiducials

For this study, a bone-anchored template embedding registration fiducials and providing an interface to fix the patient tracker rigidly was developed. A similar concept—a mouthpiece combining registration fiducials and means for patient tracking in one structure—was proposed by Labadie et al. in 2004 (40). While the concept can potentially exploit information about the predefined fiducial configuration to improve registration accuracy, it has the inherent disadvantage that displacement of the structure requires reimaging. During the third participant the tripod eventually became loose and fell off. Re-fixation and image acquisition would have unduly prolonged surgery and was not included in the study protocol. Reengineering of the fixation mechanism is required to make it more robust, potentially through a task-specific bone screw. Alternatively, for transmastoidal procedures, bone anchored screws can be placed within the retroauricular incision, and the patient tracker can be fixed to the patient's skull separately.

### Limitations

The developed freehand stereotactic image-guidance system was not used in any lateral skull base procedure. The operations performed were surgical procedures in the tympanomastoid compartment of the temporal bone (cholesteatoma, mastoid segment facial nerve schwannoma, revision cochlear implantation). Further surgeries including lateral skull base procedures (1 × schwannoma of the vestibular nerve, 1 × schwannoma of the cochlear nerve with intracochlear extension, 4 × cholesteatoma, 2 × cochlear implantation) were planned and had to be canceled at short notice due to the corona pandemic 2019-2020. The consequently limited sample size of 3 clinical cases does not allow statistical generalizations. Compared to surgery of the tympanomastoid compartment, procedures to the lateral skull base are more invasive and subject to higher risk of iatrogenic injury. Therefore, stereotactic image-guidance could have a particularly tangible effect on safety (e.g., reduction of the risk of iatrogenesis) and efficacy (e.g., reduction of the rate of tumor recurrence) for these procedures.

The proposed method for assessing usefulness and accuracy of freehand image-guidance dedicated to neurotology, while promising, has its limitations. The image pairs depict snapshots of a dynamic surgical procedure. Since only the operating surgeon has experienced the performance and information display of the device during surgery, she or he can more reliably assess its usefulness than surgeons who assess usefulness post-operatively based on static images. Because the images represent challenging but familiar clinical situations, it is assumed that domain experts can also make an assessment of usefulness postoperatively based on images, albeit with less credibility. Thus, with this method, a usefulness assessment can be obtained from multiple independent domain experts, resulting in a more objective assessment.

## Conclusion

Supporting neurotologic surgery with sufficiently accurate (μ_TRE_ + 3σ_TRE_ <0.5 mm) freehand stereotactic image-guidance is technically and clinically feasible. The use of the technology for anatomy localization and instrument navigation during neurotologic surgery is safe and useful. Preliminary results from clinical application indicate sufficient accuracy and usefulness of the presented technology for surgery of the tympanomastoid compartment of the temporal bone. Although not clinically tested, the preclinical results and the results from clinical application in tympanomastoid surgery suggest that the conclusion applies generally to neurotologic surgery.

The clinical accuracy validation via surgeon rating of corresponding images from the microscope/endoscope and image-guidance system proved purposeful. It provides a method for clinical accuracy validation of systems with expected sub-half-millimeter accuracy.

The use of a task-specific technical measurement phantom made of carbon fiber allows preclinical assessment of the error of freehand stereotactic image-guidance dedicated to neurotologic surgery with a sufficiently low measurement error (≲ 0.01 mm).

## Data Availability Statement

The original contributions presented in the study are included in the article/Supplementary Material, further inquiries can be directed to the corresponding author.

## Ethics Statement

The studies involving human participants were reviewed and approved by Cantonal Ethics Committee Bern. The patients/participants provided their written informed consent to participate in this study.

## Author Contributions

DS, SWeb, MC, and LA created the study design. DS, FM, and JH developed the hardware and software components. DS, FM, JH, and GO'TBB carried out the preclinical experiments. LA, GM, FW, SWed, MC, JH, FM, and DS carried out the clinical study. DS, LA, GM, FW, MC, SWed, GO'TBB, and SWeb analyzed the collected data. DS, FM, JH, GO'TBB, and LA wrote the manuscript. All authors reviewed the manuscript and approved the submitted version.

## Funding

This work was supported by the Swiss National Science Foundation SNF (Project 176007).

## Conflict of Interest

SW is cofounder, shareholder, and chief executive officer of CASCINATION AG Bern, Switzerland. The remaining authors declare that the research was conducted in the absence of any commercial or financial relationships that could be construed as a potential conflict of interest.

## Publisher's Note

All claims expressed in this article are solely those of the authors and do not necessarily represent those of their affiliated organizations, or those of the publisher, the editors and the reviewers. Any product that may be evaluated in this article, or claim that may be made by its manufacturer, is not guaranteed or endorsed by the publisher.
