# Supplementary material for: Freehand Stereotactic Image-Guidance Tailored to Neurotologic Surgery
Source: Front Surg. 2021 Oct 7;8:742112. doi: 10.3389/fsurg.2021.742112 (PMC8529212; doi:10.3389/fsurg.2021.742112)
Supplement: Supplementary file 1 [file Data_Sheet_1.PDF]

Cochleariform process

Participant 01

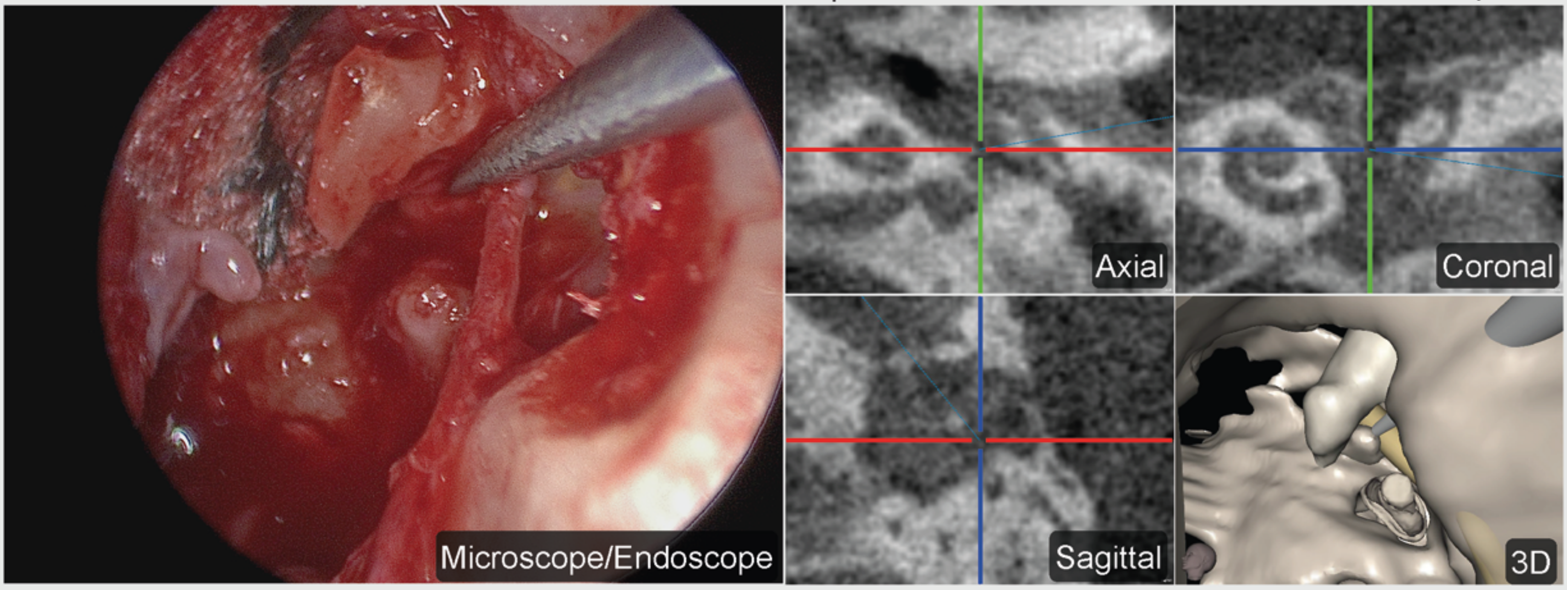

Accuracy 5 (4.5-5 , N=5)
Usefulness 4 (3-4.5, N=5)

1: Strongly disagree 2: Disagree 3: Undecided 4: Agree 5: Strongly agree

Eustachian tube

Participant 01

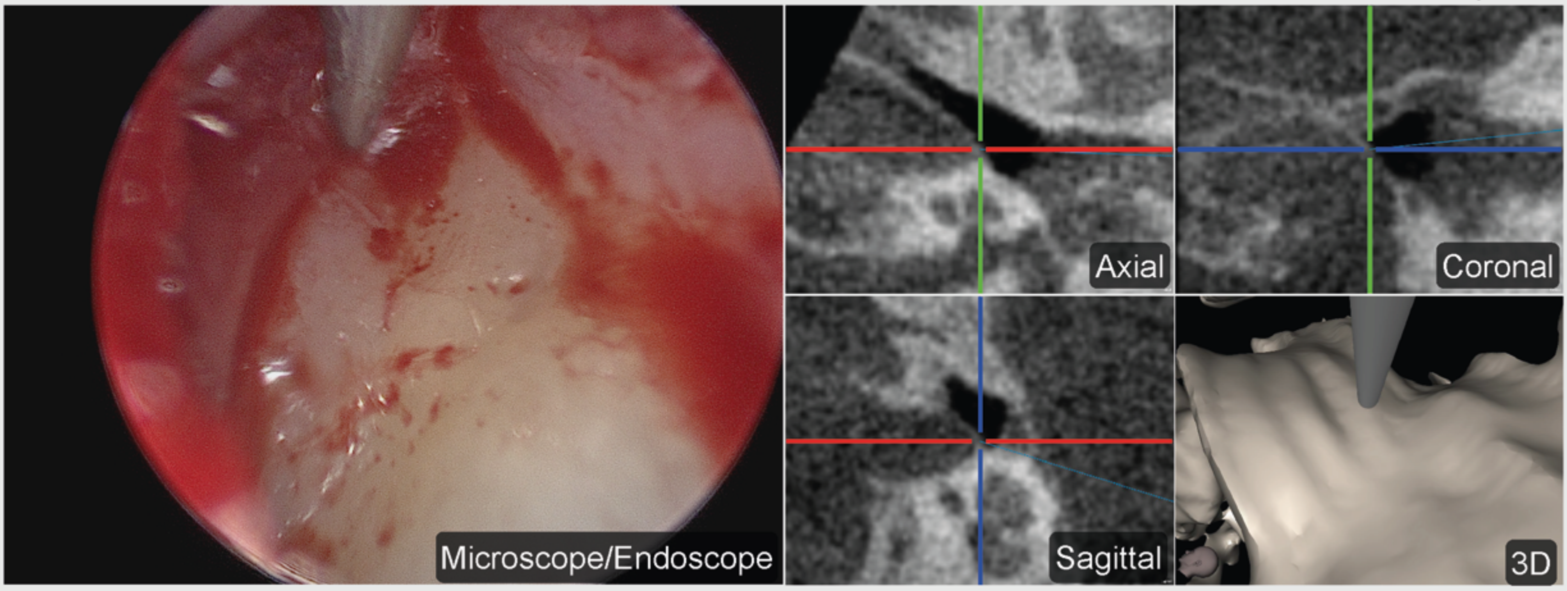

Accuracy 4.5(3.5-5 , N=5)
Usefulness 4 (2.5-4.5, N=5)

1: Strongly disagree 2: Disagree 3: Undecided 4: Agree 5: Strongly agree

Facial nerve

Participant 01

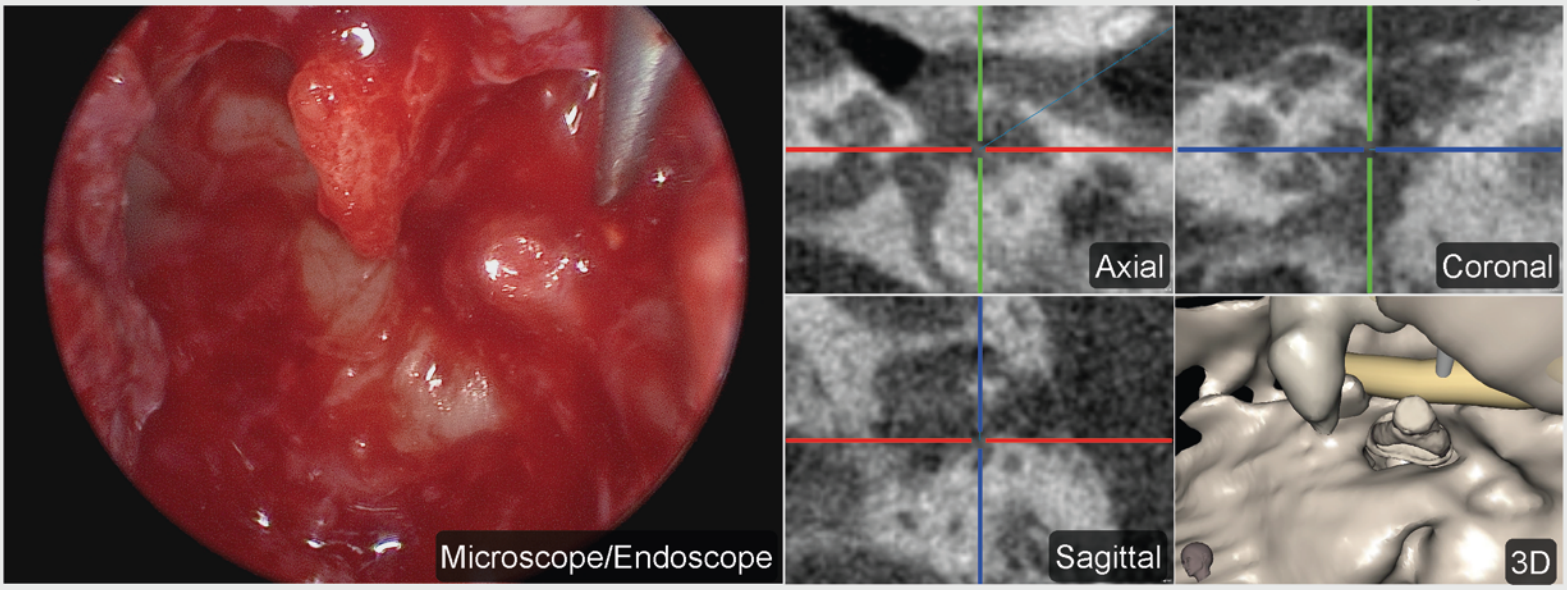

Accuracy 4 (4-5 , N=5)
Usefulness 4 (4-5 , N=5)

1: Strongly disagree 2: Disagree 3: Undecided 4: Agree 5: Strongly agree

Registration fiducial

Participant 01

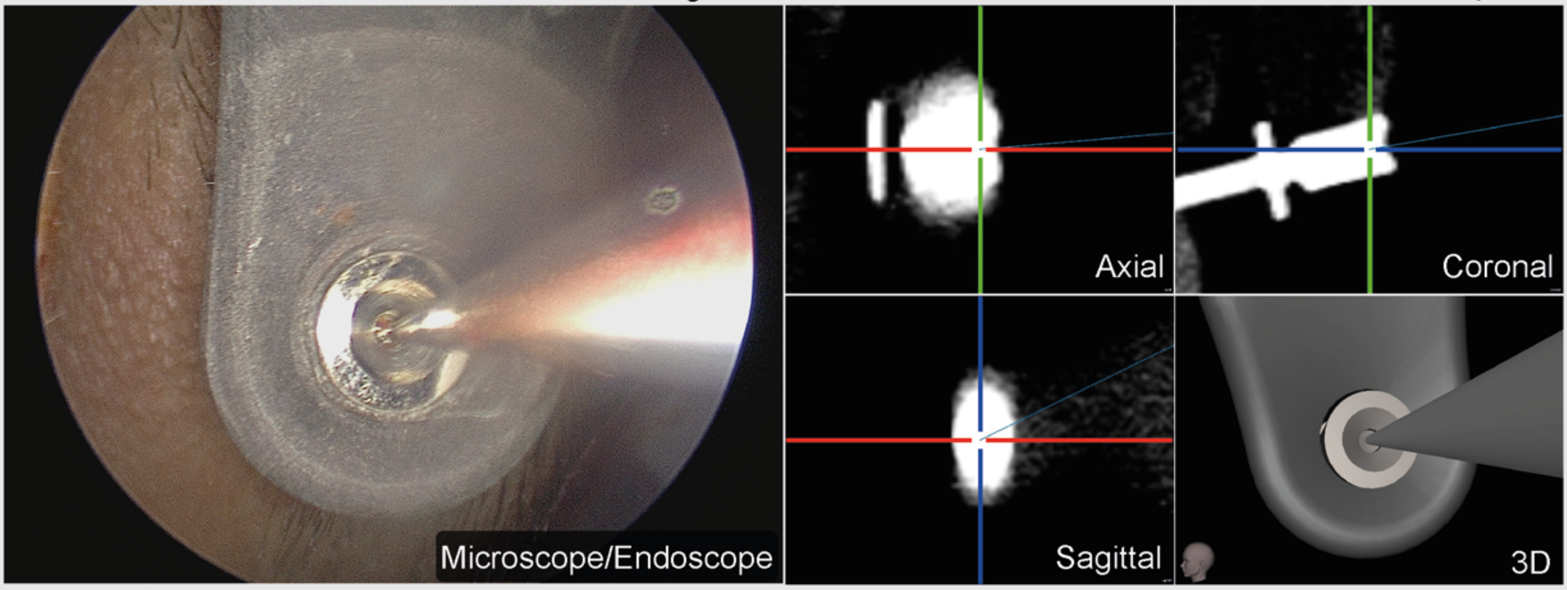

Accuracy 5 (5-5 , N=5)
Usefulness 5 (1.5-5 , N=5)

1: Strongly disagree 2: Disagree 3: Undecided 4: Agree 5: Strongly agree

Promontory

Participant 01

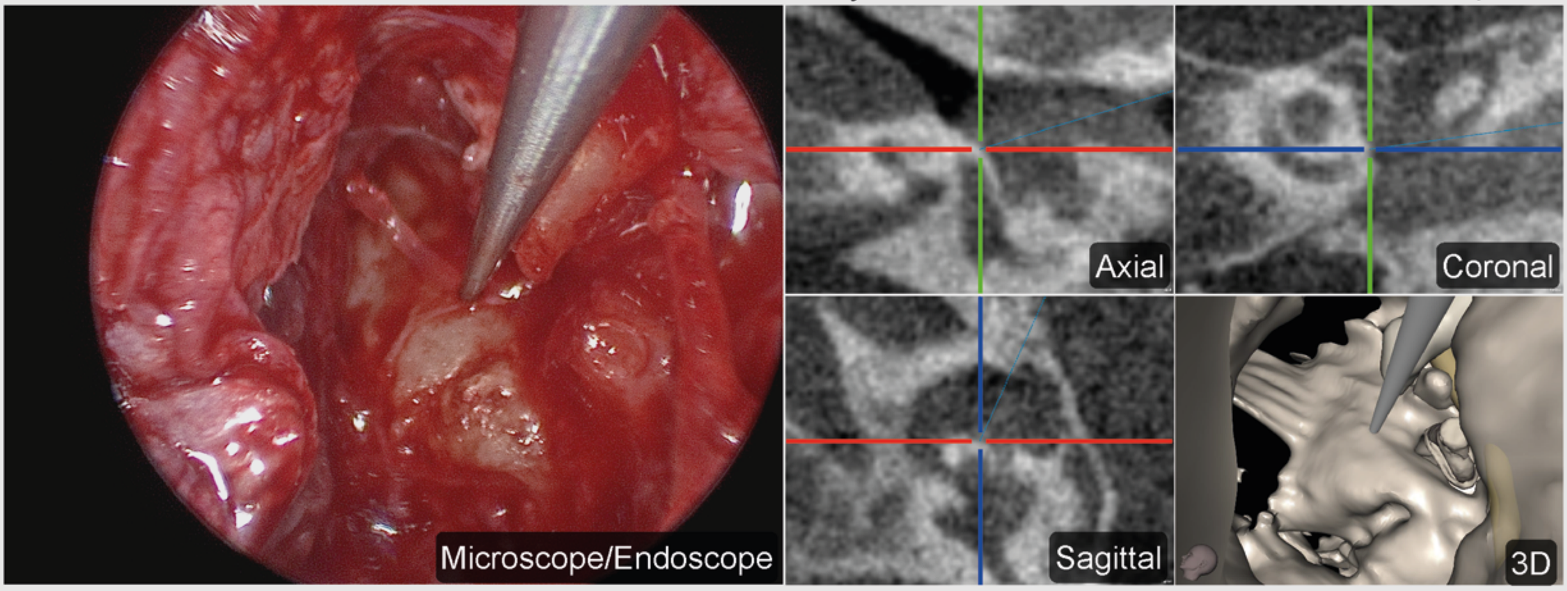

Accuracy 4 (4-5 , N=5)
Usefulness 4 (2.5-5 , N=5)

1: Strongly disagree 2: Disagree 3: Undecided 4: Agree 5: Strongly agree

Anterior pillar of round window

Participant 01

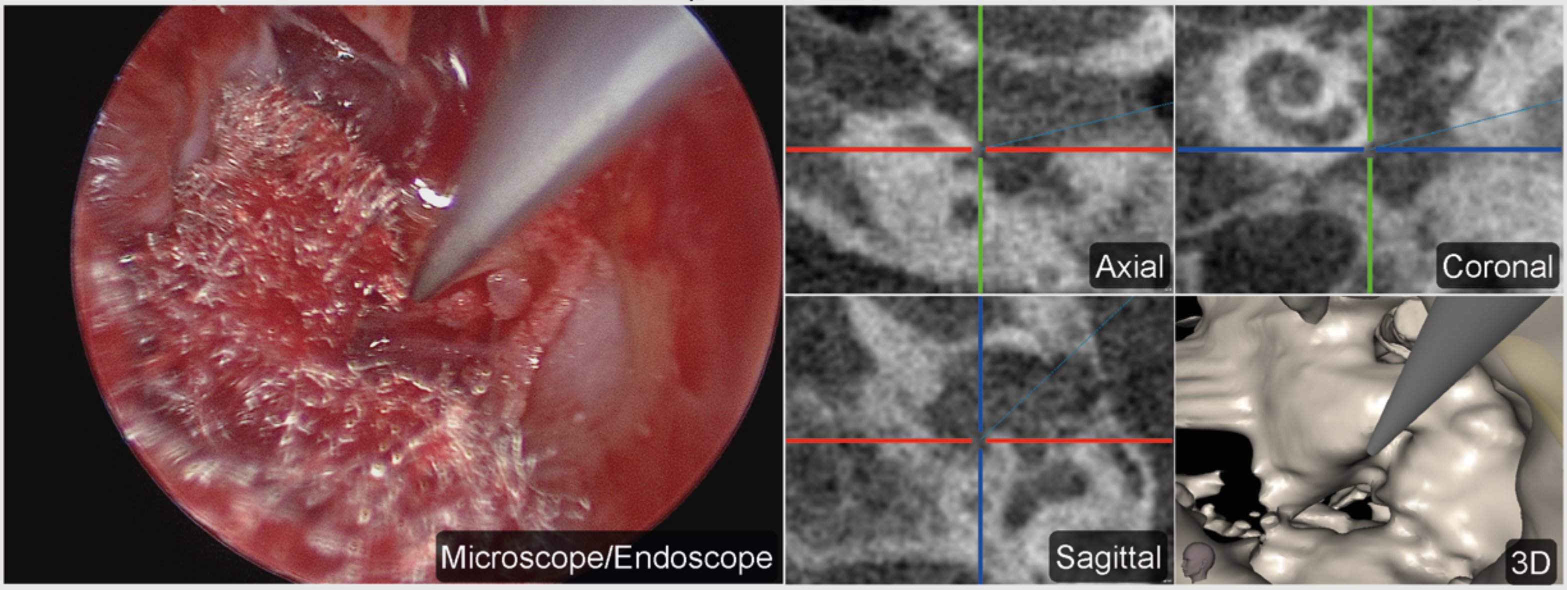

Accuracy 4 (4-5 , N=5)
Usefulness 4 (4-5 , N=5)

1: Strongly disagree 2: Disagree 3: Undecided 4: Agree 5: Strongly agree

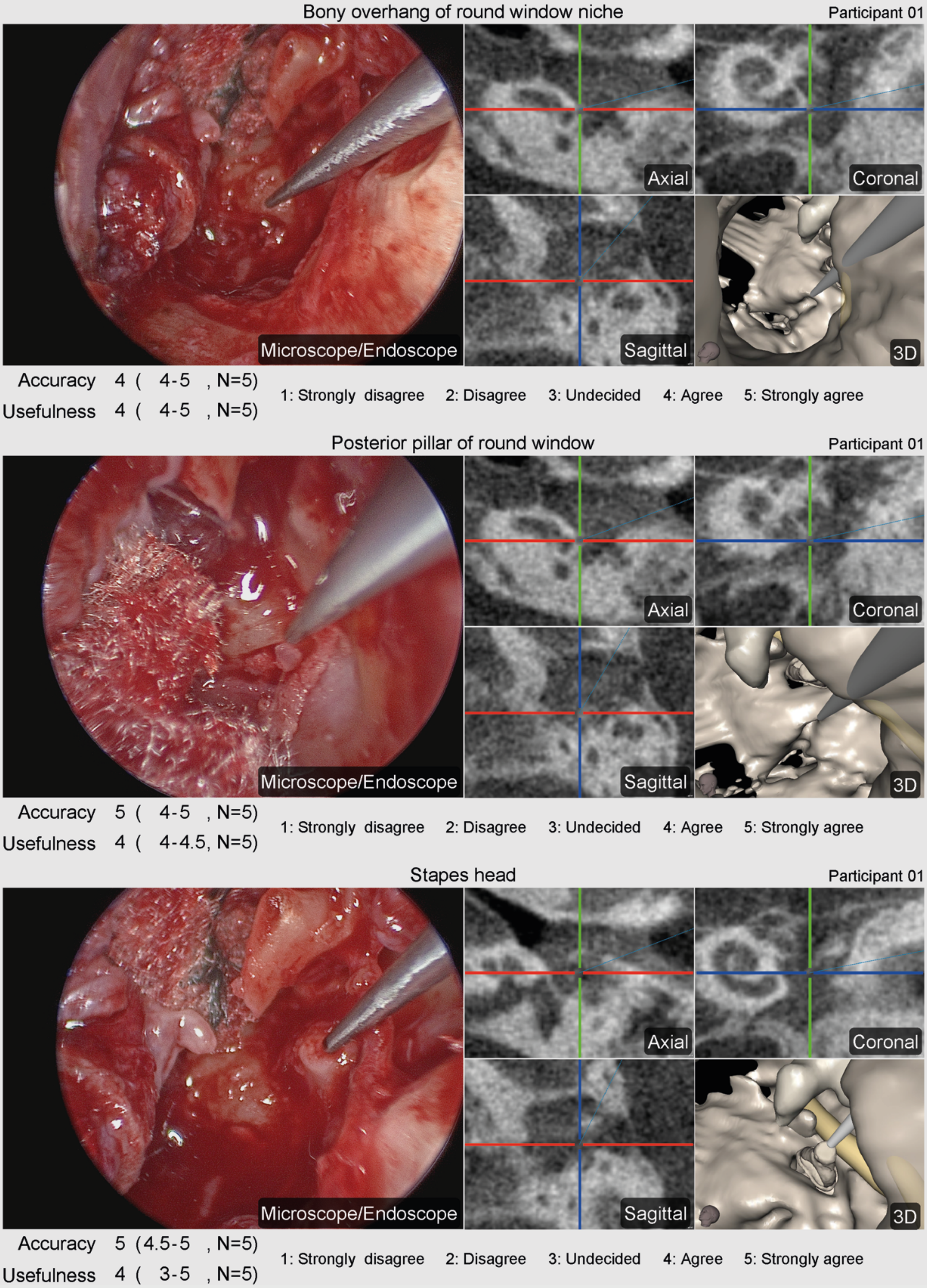
Bony overhang of round window niche
Participant 01
Axial
Coronal
Sagittal
3D
Microscope/Endoscope
Accuracy 4 (4-5 , N=5)
Usefulness 4 (4-5 , N=5)
1: Strongly disagree 2: Disagree 3: Undecided 4: Agree 5: Strongly agree
Posterior pillar of round window
Participant 01
Axial
Coronal
Sagittal
3D
Microscope/Endoscope
Accuracy 5 (4-5 , N=5)
Usefulness 4 (4-4.5, N=5)
1: Strongly disagree 2: Disagree 3: Undecided 4: Agree 5: Strongly agree
Stapes head
Participant 01
Axial
Coronal
Sagittal
3D
Microscope/Endoscope
Accuracy 5 (4.5-5 , N=5)
Usefulness 4 (3-5 , N=5)
1: Strongly disagree 2: Disagree 3: Undecided 4: Agree 5: Strongly agree

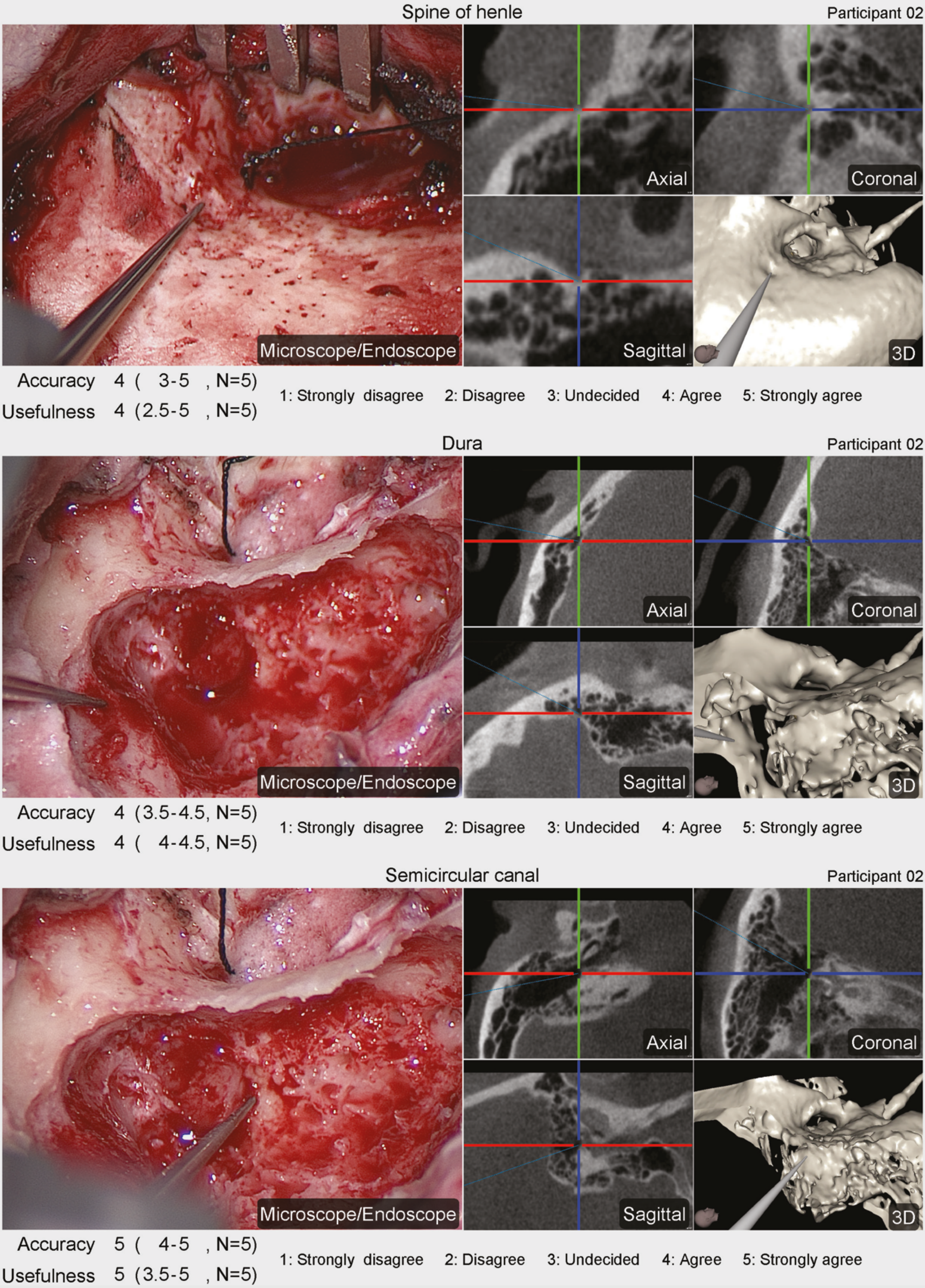

Spine of henle
Participant 02
Axial
Coronal
Microscope/Endoscope
Sagittal
3D
Accuracy 4 (3-5 , N=5)
Usefulness 4 (2.5-5 , N=5)
1: Strongly disagree 2: Disagree 3: Undecided 4: Agree 5: Strongly agree
Dura
Participant 02
Axial
Coronal
Microscope/Endoscope
Sagittal
3D
Accuracy 4 (3.5-4.5, N=5)
Usefulness 4 (4-4.5, N=5)
1: Strongly disagree 2: Disagree 3: Undecided 4: Agree 5: Strongly agree
Semicircular canal
Participant 02
Axial
Coronal
Microscope/Endoscope
Sagittal
3D
Accuracy 5 (4-5 , N=5)
Usefulness 5 (3.5-5 , N=5)
1: Strongly disagree 2: Disagree 3: Undecided 4: Agree 5: Strongly agree

Eustachian tube center

Participant 01

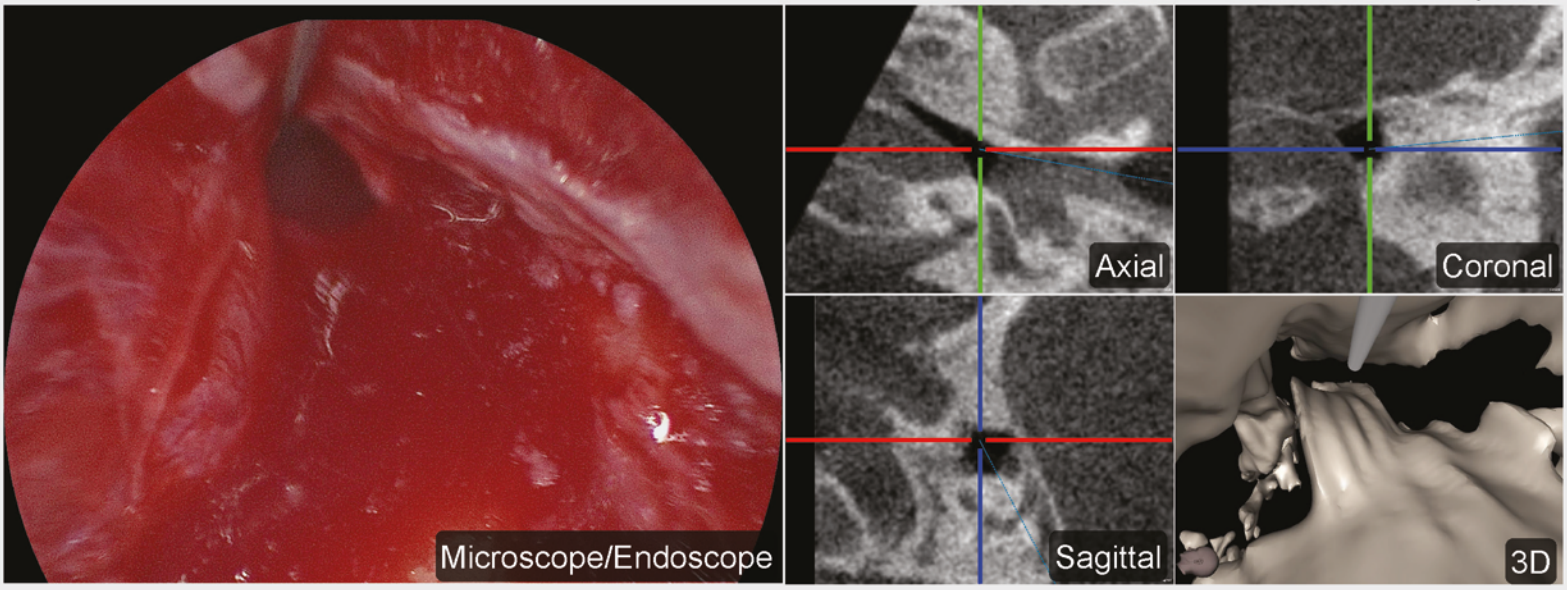

Accuracy 5 (5-5 , N=5)

Usefulness 4.5(3-5 , N=5)

1: Strongly disagree 2: Disagree 3: Undecided 4: Agree 5: Strongly agree

Digastric ridge

Participant 02

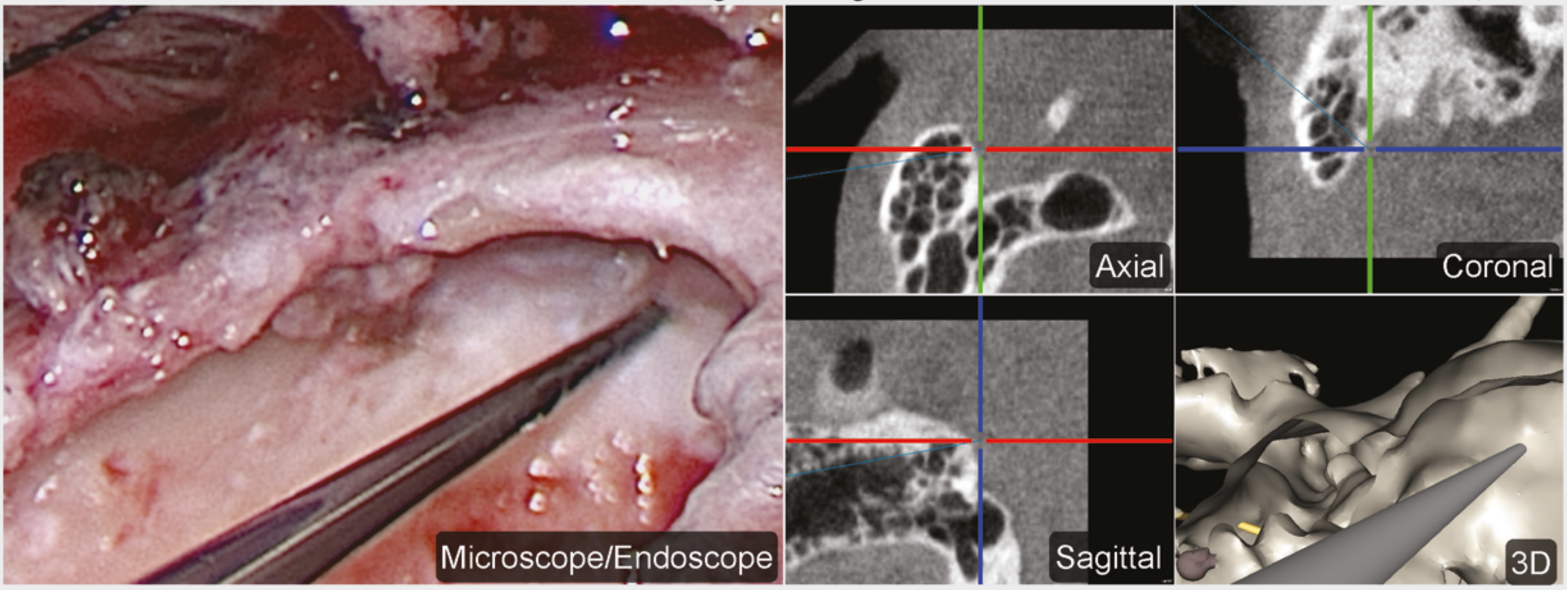

Accuracy 4 (2.5-4 , N=5)

Usefulness 4 (3.5-5 , N=5)

1: Strongly disagree 2: Disagree 3: Undecided 4: Agree 5: Strongly agree

Bony overhang of round window niche

Participant 02

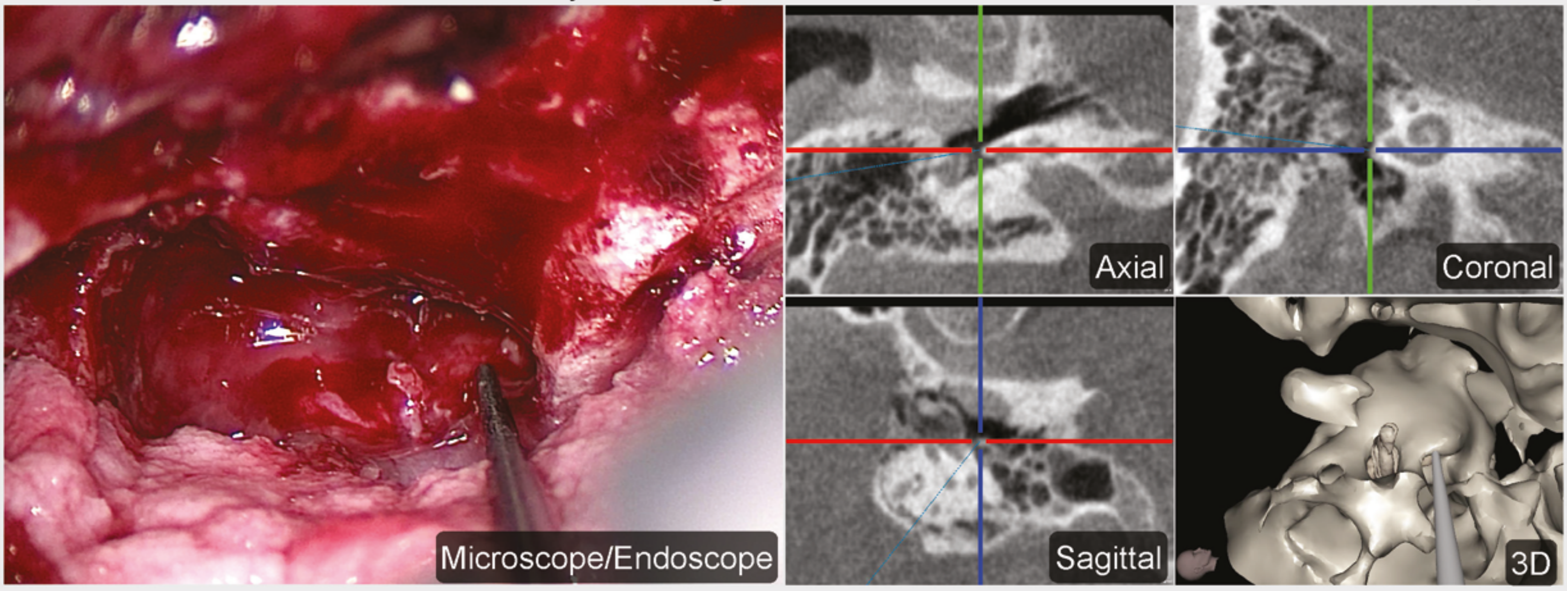

Accuracy 5 (4-5 , N=5)

Usefulness 4 (4-4.5, N=5)

1: Strongly disagree 2: Disagree 3: Undecided 4: Agree 5: Strongly agree
